# Implementation-Independent Representation for Deep Convolutional Neural Networks and Humans in Processing Faces

**DOI:** 10.3389/fncom.2020.601314

**Published:** 2021-01-26

**Authors:** Yiying Song, Yukun Qu, Shan Xu, Jia Liu

**Affiliations:** ^1^Beijing Key Laboratory of Applied Experimental Psychology, Faculty of Psychology, Beijing Normal University, Beijing, China; ^2^State Key Laboratory of Cognitive Neuroscience and Learning, Beijing Normal University, Beijing, China; ^3^Department of Psychology & Tsinghua Laboratory of Brain and Intelligence, Tsinghua University, Beijing, China

**Keywords:** deep convolutional neural network, face recognition, reverse correlation analysis, face representation, visual intelligence

## Abstract

Deep convolutional neural networks (DCNN) nowadays can match human performance in challenging complex tasks, but it remains unknown whether DCNNs achieve human-like performance through human-like processes. Here we applied a reverse-correlation method to make explicit representations of DCNNs and humans when performing face gender classification. We found that humans and a typical DCNN, VGG-Face, used similar critical information for this task, which mainly resided at low spatial frequencies. Importantly, the prior task experience, which the VGG-Face was pre-trained to process faces at the subordinate level (i.e., identification) as humans do, seemed necessary for such representational similarity, because AlexNet, a DCNN pre-trained to process objects at the basic level (i.e., categorization), succeeded in gender classification but relied on a completely different representation. In sum, although DCNNs and humans rely on different sets of hardware to process faces, they can use a similar and implementation-independent representation to achieve the same computation goal.

## Introduction

In recent years, deep convolutional neural networks (DCNN) have made dramatic progresses to achieve human-level performances in a variety of challenging complex tasks, especially visual tasks. For example, DCNNs trained to classify over a million natural images can match human performance on object categorization tasks (Krizhevsky, 2014; Simonyan and Zisserman, 2015; Krizhevsky et al., 2017), and DCNNs trained with large-scale face datasets can approach human-level performance in face recognition (Taigman et al., 2014; Parkhi et al., 2015; Schroff et al., 2015; Ranjan et al., 2017). However, these highly complex networks have remained largely opaque, whose internal operations are poorly understood. Specifically, it remains unknown whether DCNNs achieve human-like performance through human-like processes. That is, do DCNNs use similar computations and inner representations to perform tasks as humans do?

To address this question, here we applied a reverse correlation approach (Ahumada and Lovell, 1971; Gold et al., 2000; Mangini and Biederman, 2004; Martin-Malivel et al., 2006), which has been widely used in psychophysical studies to infer internal representations of human observers that transform inputs (e.g., stimuli) to outputs (e.g., behavior performance). This data-driven method allows an unbiased estimate of what is in observers' “mind” when performing a task, rather than manipulating specific features that researchers *a priori* hypothesize to be critical for the task. Here we applied this approach to both DCNNs and human observers to investigate whether the DCNNs and humans utilized similar representations to perform the task of face gender classification.

Specifically, a gender-neutral template face midway between the average male and the average female faces was superimposed with random noises, which rendered the template face more male-like in some trials or more female-like in other trials. The noisy faces were then submitted to human observers and the VGG-Face, a typical DCNN pre-trained for face identification (Parkhi et al., 2015). Based on the output of an observer that a noisy face was classified as a male but not as a female, for example, we reasoned that the noise superimposed on the template face contained features matching the observer's internal male prototype. Therefore, the difference between noise patterns of trials classified as male and those as female revealed the facial features diagnostic for gender classification, and provided an explicit and unbiased estimate of the representation used by the observer for gender classification. Finally, we directly compared the similarity of the inner representations of human observers and the VGG-Face obtained from identical stimuli and procedures, and examined the hypothesis that different intelligent information-processing systems may use similar representations to achieve the same computation goal (Marr, 1982).

## Results

### The VGG-Face and Humans Utilized Similar Information for Gender Classification

**Figure 1:**
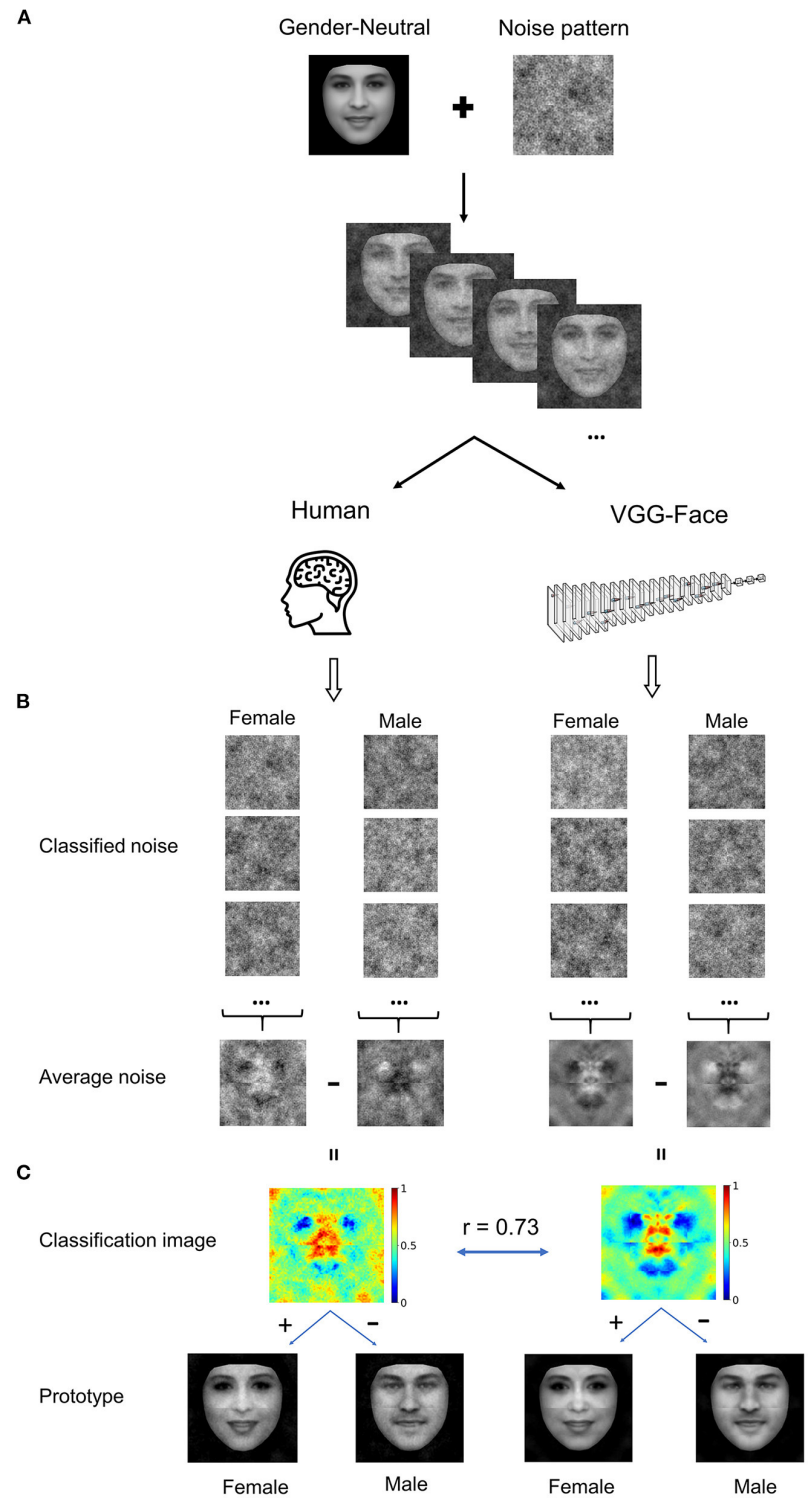
**(A)** Experiment procedure. A gender-neutral template face was superimposed with noises to create a set of gender-ambiguous faces, which were submitted to the VGG-Face and human observers for gender classification. **(B)** Exemplars of noises extracted from noisy faces classified as either female or male, respectively. The noises were then averaged to reconstruct images that contained the critical information for classifying the noisy faces as male or as female. **(C)** Classification images (CI) were the difference of the average noise of female by that of male. For visualization, values in each CI were normalized separately to the range from 0 to 1, denoted by colors. By adding or subtracting the rescaled CI to or from the gender-neutral template face, female or male prototype of human observers (Left), and the VGG-Face (Right) were created. Brain icon made by Smashicons from www.flaticon.com.

We used the reverse correlation approach to reconstruct the inner representations used by the DCNN and human observers for gender classification. Specifically, both the DCNN and human observers were asked to classify noisy faces from a gender-neutral template face embedded with random sinusoid noises as male or female (Figure 1A).

For the DCNN, we first trained the VGG-Face to classify gender using transfer learning with 21,458 face images of 52 identities (35 males) from the VGGFace2 dataset (see Methods), and the test accuracy of gender classification of the new network achieved 98.6% (see Supplementary Tables 1, 2 for more details). The gender-neutral template face was roughly equally classified as male and female by the VGG-Face (female: 54%). The noise patterns were constructed from 4,092 sinusoids at five spatial scales, six orientations, and two phases. We presented the template face embedded in 20,000 noise patterns to the VGG-Face, of which 11,736 (58.7%) images were classified as male and 8,264 (41.3%) images as female. The noise patterns from trials classified as male or female were averaged separately (Figure 1B), and the difference between the two average noise patterns yielded a “classification image” (CI) that makes explicit the information used by the VGG-Face for gender classification (Figure 1C). A visual inspection of the CI showed that regions around the eyes, nose, and mouth were of high contrast in the CI, indicating the critical regions employed by the VGG-Face to classify male from female faces.

Then, we reconstructed the representation used by human observers in a similar way. In our study, 16 human observers performed the gender classification task, each presented with 1,000 noisy faces. Altogether, 16,000 images were presented to the human observers, of which 7,969 (49.8%) images were classified as males and 8,031 (50.2%) images as females. Similarly, the CI for human observers was obtained (Figure 1C). Visual inspections of the CIs for the VGG-Face and human observers revealed good agreement between them, and Pearson's correlation between the two CIs was high (*r* = 0.73). This result suggested that the VGG-Face and human observers utilized similar information to classifying gender.

Further, we reconstructed inner male and female prototypes by adding or subtracting the rescaled CI to or from the template face for the VGG-Face and humans, respectively (Figure 1C). As expected, the male and female prototype faces are perceptually male-like and female-like, and highly similar between the VGG-Face and human observers.

### The Shared Representation Was Mainly Based on Low Spatial-Frequency Information

**Figure 2:**
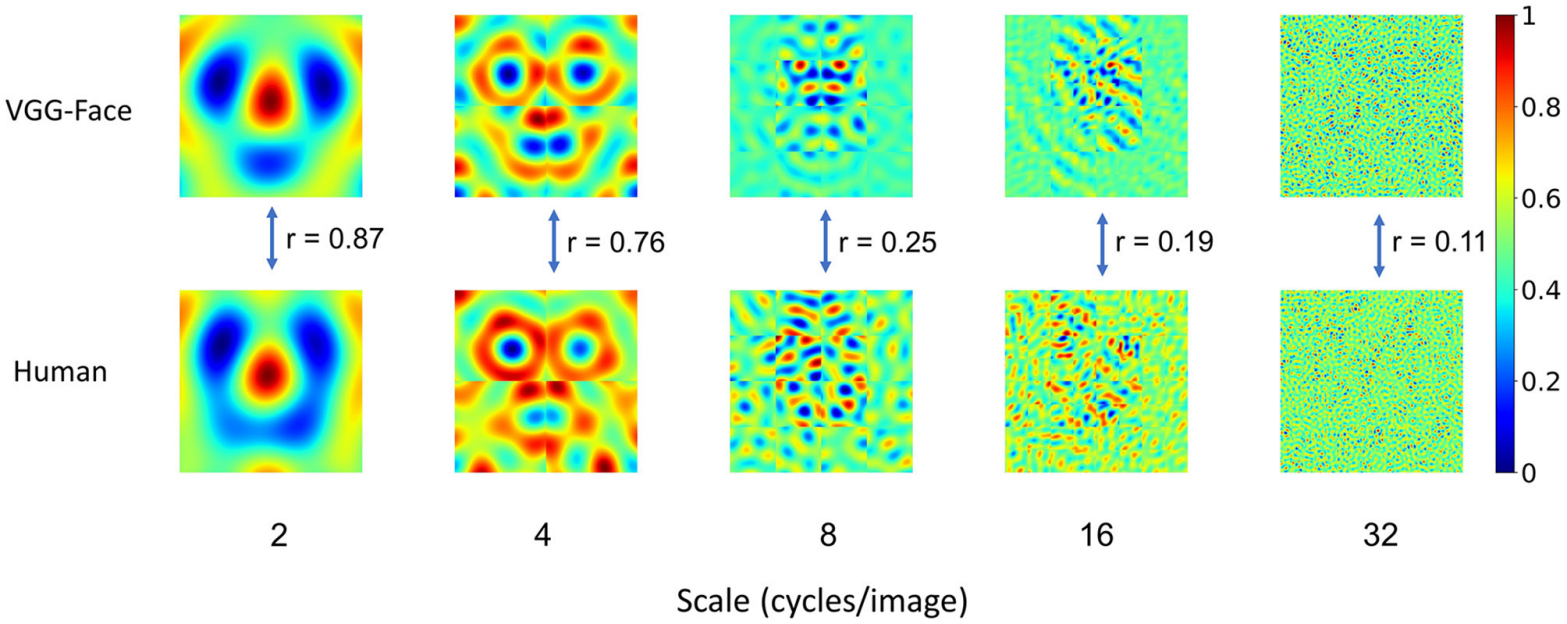
Correspondence in representation at different scales of spatial frequencies. For visualization, values in each CI were normalized separately to the range from 0 to 1, denoted by colors. Note that the correspondence was the highest at the low-spatial frequencies, and then decreased sharply at the high-spatial frequencies. Scale number denotes cycles per image.

Having found that the VGG-Face and human observers utilized similar information for gender classification, next we asked whether the VGG-Face and human observers employed similar information in all spatial frequencies. In our study, the noise patterns were constructed from sinusoid components of five scales of spatial frequencies (2, 4, 8, 16, and 32 cycles/image), which enabled us to reconstruct the CIs for each scale separately (Figure 2) and examined the similarity at each scale. We found that the similarity was the highest at low spatial frequencies (*r* = 0.87 and 0.76 at 2 and 4 cycles/images), and then decreased sharply at high spatial frequencies (*r* = 0.25, 0.19, 0.11 at 8, 16, and 32 cycles/image). Consequently, male and female prototypes reconstructed with the noise patterns at low spatial frequencies (2 and 4 cycles/image) were more similar between human observers and the VGG-Face than those at high spatial frequencies (8, 16, and 32 cycles/images) (Supplementary Analysis 1). Therefore, the shared representation for gender classification was mainly based on information at low spatial frequencies, consistent with previous findings that face gender processing relies heavily on low spatial frequencies (Sergent, 1986; Valentin et al., 1994; Goffaux et al., 2003b; Mangini and Biederman, 2004; Khalid et al., 2013).

**Figure 3:**
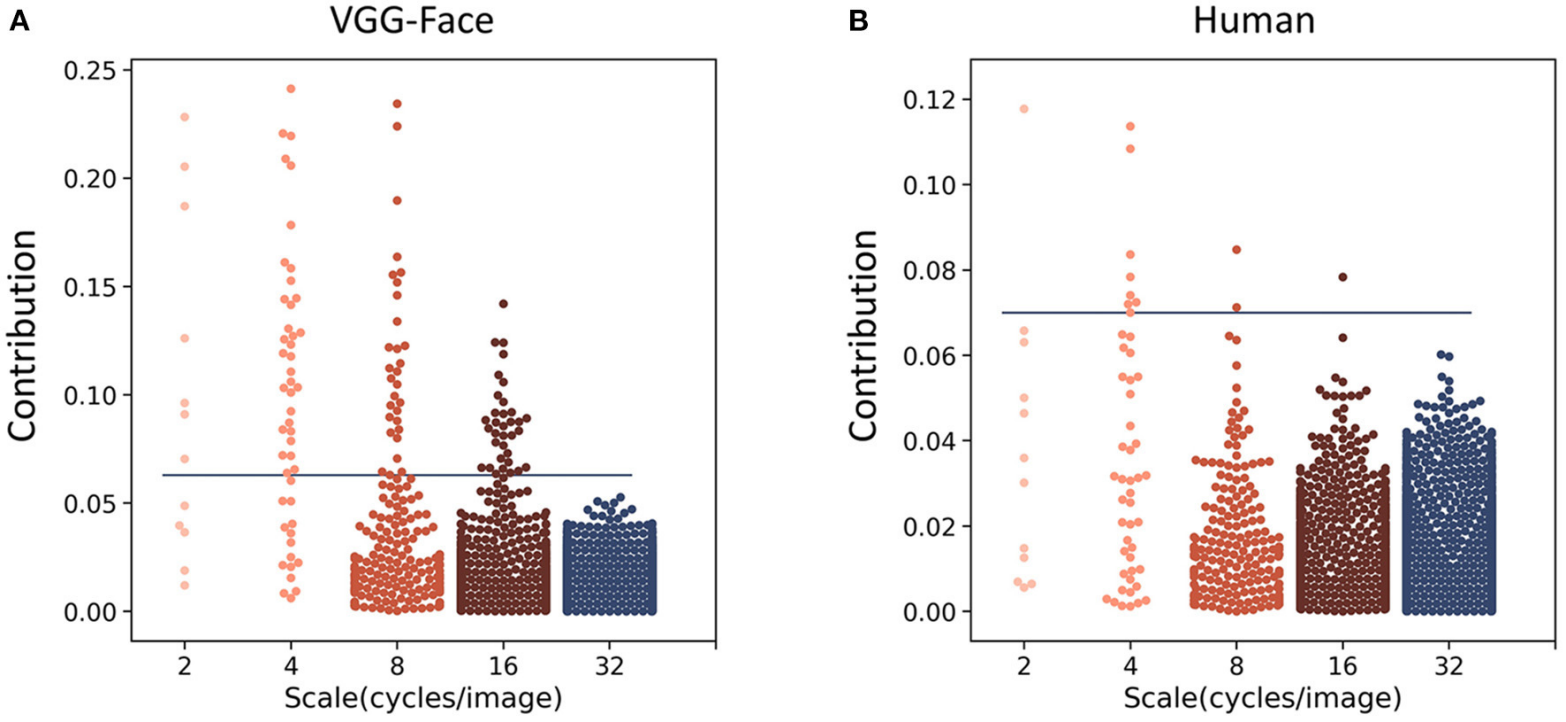
Manhattan plot of the contribution (the absolute values of Cohen's d) of the parameters used to construct noises in the VGG-Face **(A)** and in human observers **(B)**. Each dot denotes a parameter, and the horizontal blue line indicates the significance level after Bonferroni correction.

To further quantify the contribution of different spatial frequencies for gender classification, we calculated the contribution of each of the 4,092 parameters from all five spatial frequencies. For each parameter, we performed an independent sample *t-*test (two-sided) between the parameter values from the male trials and those from the female trials, and calculated the absolute value of Cohen's d as an index of the contribution of each parameter to gender classification. One hundred and four parameters in the VGG-Face and 12 in human observers contributed significantly for the classification (Bonferroni corrected for multiple comparisons, Figures 3A,B). Of the 12 parameters in human observers, 9 were at the scales of 2 and 4 cycles/images. Similarly, most of the 104 parameters in the VGG-Face were also at low-frequency scales (7 at 2 cycles/images, 33 at 4 cycles/images, and 30 at 8 cycles/images), and the percentage of the significant parameters at low frequencies (58 and 69% at 2 and 4 cycles/images) were much higher than those at high frequencies (4 and 0% at 16 and 32 cycles/images). That is, both the VGG-Face and human observers mainly relied on information at low spatial frequencies for gender classification.

Another way is to select parameters that made the most contributions indexed by the absolute values of Cohen's d. We found that the 1,885 most contributing parameters of all 4,092 parameters already made up to 80% of the total contribution for the VGG-Face; importantly, these parameters also made up 48% of the contribution for human observers. Then, we examined the similarity of parameters' contribution by calculating the Spearman's correlation between Cohen's d of the VGG-Face and human observers for the highly-contributing parameters at each scale of spatial frequencies. We found that the correlation was high at low spatial frequencies (*r* = 0.79 and 0.74 at 2 and 4 cycles/images), and then declined sharply at high spatial frequencies (*r* = 0.21, 0.27, and 0.17 at 8, 16, and 32 cycles/images). In contrast, there were more parameters at high than low spatial frequencies that contributed differently between the VGG-Face and human observers (Supplementary Analysis 2). Taken together, at low spatial frequencies, not only were the representations more similar, but also the parameters underlying the representation contributed more significantly to the task.

### Human-Like Representation Requires Prior Experience of Face Identification

**Figure 4:**
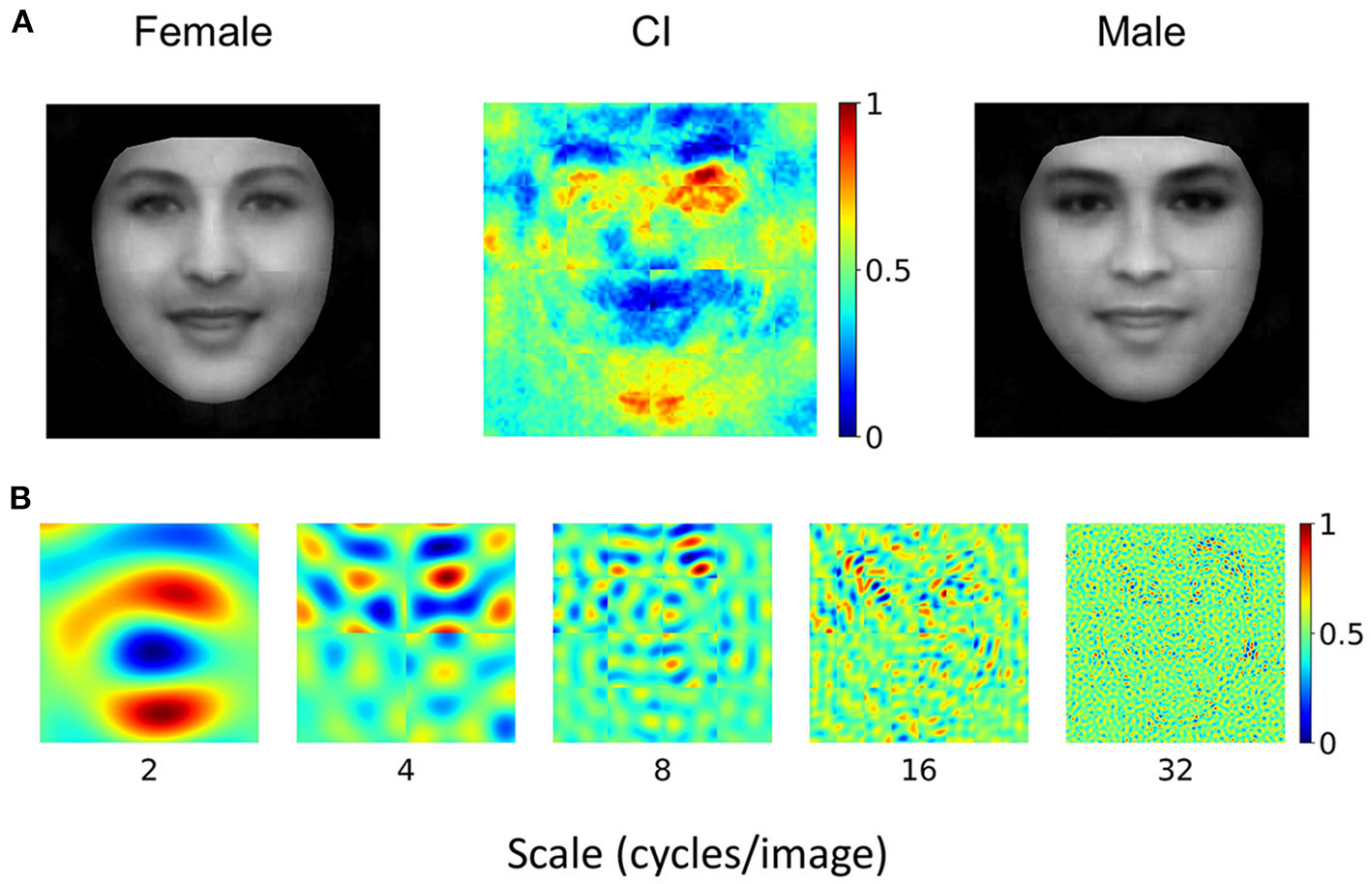
**(A)** AlexNet's CI for gender classification. For visualization, values in each CI were normalized separately to the range from 0 to 1, denoted by colors. Note that the female prototype (left) and the male prototype (right) were not perceptually female-like and male-like, respectively. **(B)** Normalized CI at different scales of spatial frequencies. Note that they were significantly different from those of human observers.

Where did the representational similarity come from? One possibility is that information at low spatial frequencies is critical for face processing, and therefore both DCNN and human observers were forced to exact information at low spatial frequencies to successfully perform the task. An alternative hypothesis is that the VGG-Face and human observers share similar prior experiences of processing face at the subordinate level where faces are identified into different individuals. To test these two hypotheses, we examined another typical DCNN, the AlexNet, that also has abundant exposure to face images but is pre-trained to classify objects into 1,000 basic categories. We trained the AlexNet to perform the gender classification task with the same transfer learning procedure as that for the VGG-Face. The testing accuracy of gender classification of the AlexNet reached 89.3% (see Supplementary Tables 1, 2 for more details), indicating that it was able to perform the task. However, the CIs obtained from the Alexnet (Figures 4A,B) were in sharp contrast to the CIs of human observers (Figures 1C, 2) as a whole (*r* = −0.04) and at different scales (*r* = −0.28, 0.03, 0.25, 0.10, and 0.03 at the scales of 2, 4, 8, 16, and 32). We also reconstructed the female and male prototype faces of AlexNet (Figure 4A), and they appeared quite distinct from those of human observers and the VGG-Face (Figure 1C). This finding was unlikely due to the differences in architecture between the VGG-Face and the AlexNet, because the VGG-16, which has the same architecture as the VGG-Face but is pre-trained for object categorization as the AlexNet, showed a CI largely different from human observers (Supplemental Analysis 3). Therefore, although the AlexNet succeeded in performing the gender classification task, it relied on a set of information completely different from human observers to achieve the goal. Therefore, mere exposure to face stimuli or large categories of stimuli is not sufficient for the DCNNs to construct similar representations for gender classification as human observers; instead, the task requirement of face identification during prior experience was required.

Given that the training sample contained more male than female faces, we also trained the VGG-Face and AlexNet for face-gender classification with balanced training sample to exclude the possibility that our results was caused by unbalanced training sample (Supplementary Analysis 4).

In addition, to examine whether our results could transfer to other face databases, we trained the VGG-Face and AlexNet for face-gender classification using face images from another database FairFace, the Face Attribute Dataset for Balanced Race, Gender, and Age (Kärkkäinen and Joo, 2019), and the main findings were replicated with this new dataset (Supplementary Analysis 5).

**Figure 5:**
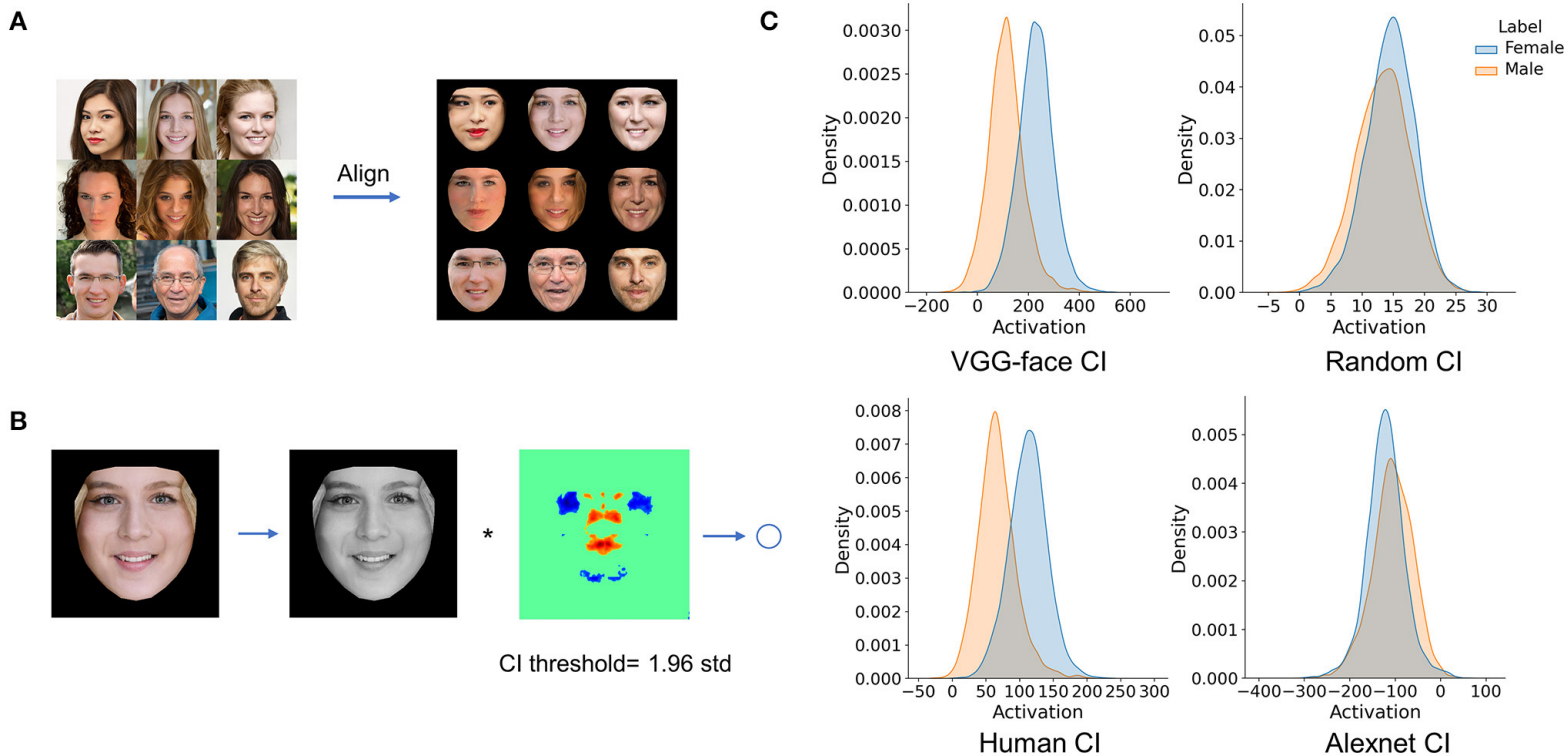
Using CIs in a simple network. **(A)** Each of 26,902 face images was aligned to a neutral face template. **(B)** Each aligned face was convolved with the CI to get an activation value. **(C)** Activation distributions of the female and males faces after convolving with the CIs.

Finally, to further illustrate that the CIs obtained here reflected representations for face gender classification, we built a simple new network that used the CIs to perform gender classification. Specifically, we first aligned each of 26,902 face images (13,738 females) to the neutral face template and convolved each aligned face with the CI to get an activation value (Figures 5A,B). This procedure is equivalent to using each aligned face as an input image and the CI as connected weights of a one-layer network with one output unit. If the CI does represent the differences between female and male faces, the activation distributions of male and female faces would be dissociated. As shown in Figure 5C, after convolving with the CI obtained from VGG-Face, the activation distribution of female faces dissociated from that of male faces (cohen's d = 1.62). A similar trend of dissociation was observed when using the CI obtained from humans (cohen's d = 1.58). As a baseline, we randomized the CI image and convolved each face with the randomized CI image, and the two activation distributions largely overlapped (cohen's d = 0.30). These results indicated that the CIs revealed the information used for face gender classification, and similar information was used by VGG-Face and humans. When using the CI obtained from AlexNet, the two activation distributions also largely overlapped (cohen's d = −0.35), and the difference between female and male activations was in an opposite direction to the results of VGG-Face and humans. Again, this result was consistent with our main finding that the CI of AlexNet differed from that of VGG-Face and humans.

## Discussion

Marr (1982) has proposed a three-level framework to understand an intelligent information-processing system. At the top is the computational level that defines the goal of the system, and in our study, the computation goal is face gender classification; at the bottom is the implementation level that is the physical substrate of the system, which are the DCNNs and human brain in our study. Most critically, in the middle is the representational and algorithmic level that establishes approaches through which the implementation achieves the computation goal. Despite dramatic differences in the physical implementations between the artificial and biological intelligent systems, similar representations may be used by different systems to achieve the same computation goal. Our study provides one of the first direct evidence to support this hypothesis by showing that the DCNNs and humans used similar representations to achieve the goal of face gender classification, which were revealed by highly similar CIs between the VGG-Face and humans. Admittedly, the present study examined face perception which is highly domain-specific in human visual cognition. Future study is needed to examine whether implementation-independent representation can also be observed in less specialized perceptual processes.

The shared representation, on one hand, may come from the critical stimulus information needed to achieve the computation goal. Previous human studies on gender classification suggest that the critical information humans used to solve the task is embedded mainly in low spatial frequencies (Sergent, 1986; Valentin et al., 1994; Goffaux et al., 2003b; Mangini and Biederman, 2004; Khalid et al., 2013). Here we found that the VGG-Face also relied heavily on low spatial frequencies of faces for gender classification. Further, it was the information only in this band that showed similarity to that of humans, but not in high spatial frequencies. In other words, one reason that the VGG-Face and humans established similar representations based on low spatial frequencies might be that this stimulus information is critical for the task of face gender classification.

On the other hand, the prior task experience before the gender classification task may also play a deterministic role for DCNNs to use a similar approach to achieve the goal as humans. Previous studies have shown that humans usually process faces at the subordinate level, that is, to recognize faces as individuals. Similar to humans, the VGG-Face is also pre-trained to recognize faces at the individual level, that is, to classify face images into different identities (e.g., John's face). Therefore, the similar task experience in the past likely led the similar approaches in achieving the new goal of gender classification.

In contrast, the AlexNet is pre-trained to recognize objects at the basic level, that is, to classify objects into categories (e.g., dogs) but not individuals (John's dog). Therefore, although the AlexNet experiences abundant exposure to face images during the pre-training, it processes faces as objects, different from humans and the VGG-Face. Previous studies on humans have shown that object recognition does not selectively rely on low- to middle- spatial frequencies as face recognition does (Biederman and Kalocsais, 1997; Goffaux et al., 2003a; Collin, 2006; Collin et al., 2012). Thus, it is not surprising that although the AlexNet also achieved a high performance (accuracy around 90%) in face gender classification, an approach significantly different from that of humans was adopted. Taken together, the similarity in representation between DCNNs and humans was not guaranteed by the common computational goal or by the passive experiences with stimuli; instead, it was constrained by the combination of experiences on the pre-training task in the past and critical stimulus information needed in performing the task in the present. The finding also suggests that DCNN can be used as a model of biological brains to experimentally investigate the effect of visual experience and task demands on human cognition.

The present study also brought insight from an engineering perspective. In history, two main approaches have been proposed to achieve and even excel human vision in artificial intelligence (Kriegeskorte and Douglas, 2018). The neuroscience approach adheres to biological fidelity at the implementation level, which simulates neural circuits of brains, whereas the cognitive approach emphasizes on cognitive fidelity, which focuses on goal-directed algorithms and disregards implementation. Our study suggests an intermediate approach lying in between these two. By simulating human intelligence at the representation level in Marr's framework, this approach provides an abstract description of how a system extracts critical features to construct representation for a specific task. Because the representation is relatively independent of implementation, the knowledge acquired in biological systems can be easily adopted by artificial systems with completely different substrates. Therefore, the simulation of representation may shed light on building new AI systems in a feasible way.

## Materials and Methods

### Transfer Learning

We used the pre-trained VGG-Face network (Parkhi et al., 2015) that consists of 13 convolutional layers and 3 fully connected (FC) layers. Each convolutional layer and FC layer were followed by one or more non-linearities such as ReLU and max pooling. The VGG-Face network was pre-trained for face identification with the VGG-Face dataset containing over two million face images of 2,662 identities.

In our study, we trained the VGG-Face for face-gender classification using transfer learning. The final FC layer of the VGG-Face has 2,662 units, each for one identity. We replaced this layer with a two-unit FC layer for the binary gender classification. All weights of the network were frozen except the weights between the penultimate FC layer and the new final FC layer. The training sample contains 21,458 face images (male: 14,586) of 52 identities (male: 35) randomly selected from the VGGFace2 dataset (Cao et al., 2018). The validation sample contains other 666 face images (male: 429) from the same 52 identities. The testing sample contains 1,000 face images (male: 500) from 24 new identities from the VGGFace2 dataset. All face images were resized to 224 × 224 pixels to match the model input size. We used in-house python package DNNbrain (Chen et al., 2020) to train the network. The loss function was cross-entropy, and the optimizer was Adam. The learning rate was 0.03, and the network was trained for 25 epochs. After training, the accuracy of gender classification reached 100% on both the training and validation samples, and 98.6% on the testing sample.

The same training procedures were applied to AlexNet pre-trained for object categorization (Krizhevsky et al., 2017). The model consists of five convolutional layers and three FC layers. The AlexNet was pre-trained on ImageNet to classify 1.2 million images into 1,000 object categories. We also replaced the final layer of AlexNet with a two-unit FC layer for the binary gender classification. After transfer learning, the accuracy for gender classification reached 92.6% on the training sample, 93.2% on the validation sample, and 89.3% on the testing sample.

### Reverse Correlation Approach

After the transfer learning on gender classification, we made the representation explicit with the reverse correlation approach on noisy faces. All stimuli consisted of a gender-neutral template face superimposed with sinusoid noise patterns. The template was a morphed face between a female average face and a male average face (Figure 1A). The female and male average faces were computed as a mathematical average of all female and all male faces of the training sample after they were aligned and wrapped into the same space with 68 landmarks using an open-access toolbox face_morpher (https://github.com/alyssaq/face_morpher). The average faces were 8-bit grayscale and 512 × 512 pixel images. We further created 500 morph faces that gradually changed from the female average face to the male average face using face_morpher. Then we presented 500 morphed faces evenly distributed between the female and the male average faces to the VGG-Face to find the face most equally classified as male and female in gender classification. The 250th morphed face, which was classified as female with a probability of 54% by the VGG-Face, was chosen as the gender-neutral template face in our study.

A random noise pattern was generated for each trial. Each noise pattern was composed of sinusoid patch layers of five different scales of spatial frequencies (2, 4, 8, 16, and 32 cycles/image), with each patch layer made up of 1, 4, 16, 64, and 256 sinusoid patches, respectively (Mangini and Biederman, 2004). For each sinusoid patch, sinusoids of six orientations (0, 30, 60, 90, 120, and 150 degrees) and two phases (0 and pi/2) were summed. The amplitude of each sinusoid came from a random sampling of a uniform distribution of values from −1 to 1. Therefore, each noise pattern was determined by 4,092 random amplitude parameters (12, 48, 192, 768, and 3,072 parameters for 2, 4, 8, 16, and 32 cycles/image). We use the R package rcicr to generate the sinusoid noises (Dotsch, 2017). We created 20,000 noise patterns for the DCNNs and 1,000 noise patterns for each human observer. Each noise pattern was then superimposed on the template face to create a different noisy face.

We resized the noisy face images to 224 × 224 pixels and submitted them to the VGG-Face and AlexNet, and obtained their classification prediction for each image. For VGG-Face, a noisy face was classified as male when the activation of the male unit was higher than the female unit. Note that the AlexNet showed a bias toward male faces when classifying the noisy faces; therefore, we modified the classification criterion for the AlexNet. That is, for AlexNet, a noisy face would be classified as male when the activation of the male unit to the to-be-classified face was higher than its average activation to all noisy faces. Note that the choice of criterion would not affect the results pattern of the VGG-Face and hence the dissociation between AlexNet and VGG-Face, because the two criteria lead to literally identical CIs for VGG-Face (*r* = 0.99).

To generate corresponding female or male prototype faces, each CI was separately rescaled to have the same maximum pixel value and then added or subtracted from the template face.

### Participants

Sixteen college students (12 females, age 19–33 years, mean age 22 years) from Beijing Normal University, Beijing, China, participated in the gender classification task. All participants were right-handed and had normal or corrected-to-normal vision. The experiment protocol was approved by the Institutional Review Board of the Faculty of Psychology, Beijing Normal University. Written informed consent was obtained from all participants before the experiment.

### Experimental Procedures

Before the experiment, participants were told that they would perform a difficult gender classification task because the faces were superimposed with heavy noises. The template image was not shown to participants in the experiment. The stimuli were 255-bit grayscale and 512 × 512 pixel images. PsychoPy (Peirce et al., 2019) was used to display the stimuli and record responses. The stimuli were presented on the screen of a Dell precision laptop at a distance of 70 cm. The stimuli subtended a visual angle of ~8.2 degree. In each trial, a noisy face image was presented in the center of the screen for 1 s, and then the screen cleared until the participant made a response. The participants were instructed to provide one of four responses with a key press for each trial: probably female, possibly female, possibly male, or probably male. No feedback was provided. Each participant performed 1,000 trials. The participants could rest every 100 trials. The total experiment duration was about 1 h for each participant. In data analysis, the CI was calculated by subtracting the average noise patterns from all trials classified as male (probably male and possibly male) from those classified as female (probably female and possibly female).

## Data Availability Statement

All codes for analyses are available on https://github.com/YukunQu/cnnface. All face images used in the present study were from the public VGGFace2 dataset and FairFace dataset. The behavioral data are available on https://osf.io/6gqtx/.

## Ethics Statement

The studies involving human participants were reviewed and approved by the Institutional Review Board of the Faculty of Psychology, Beijing Normal University. The patients/participants provided their written informed consent to participate in this study.

## Author Contributions

JL and YS conceived and designed the study. YQ collected and analyzed the data. YS interpreted the data and wrote the manuscript with input from JL, YQ, and SX. All authors contributed to the article and approved the submitted version.

## Conflict of Interest

The authors declare that the research was conducted in the absence of any commercial or financial relationships that could be construed as a potential conflict of interest.
