# Supplementary material for: Implementation-Independent Representation for Deep Convolutional Neural Networks and Humans in Processing Faces
Source: Front Comput Neurosci. 2021 Jan 26;14:601314. doi: 10.3389/fncom.2020.601314 (PMC7870475; doi:10.3389/fncom.2020.601314)
Supplement: Supplementary file 1 [file Image_1.PDF]

Supplementary Material

Supplementary analysis 1

To investigate how different scales of spatial frequencies affected gender classification, we constructed male and female prototypes for each spatial frequency respectively. Visual inspection of Figure S1a revealed that the prototype images at 2 and 4 cycles/images were highly similar between the VGG-Face and human observers, while those at 8 and 16 cycles/images showed noticeable differences. To validate this intuition, we presented these prototype images to the VGG-Face, and examined whether they elicited similar activations in the final fully-connected layer. We found that for the scales at low spatial frequencies (2 and 4 cycles/images), the prototype images obtained from the VGG-Face and human observers elicited similar activation amplitudes, while at the scales of high spatial frequencies (8, 16, and 32 cycles/images), the prototype images obtained from the VGG-Face elicited much higher activation amplitudes than those obtained from humans (Figure S1b). Taken together, the VGG-Face and human observers shared similar inner representations at low spatial frequencies, and the similarity decreased along with the increase of spatial frequencies

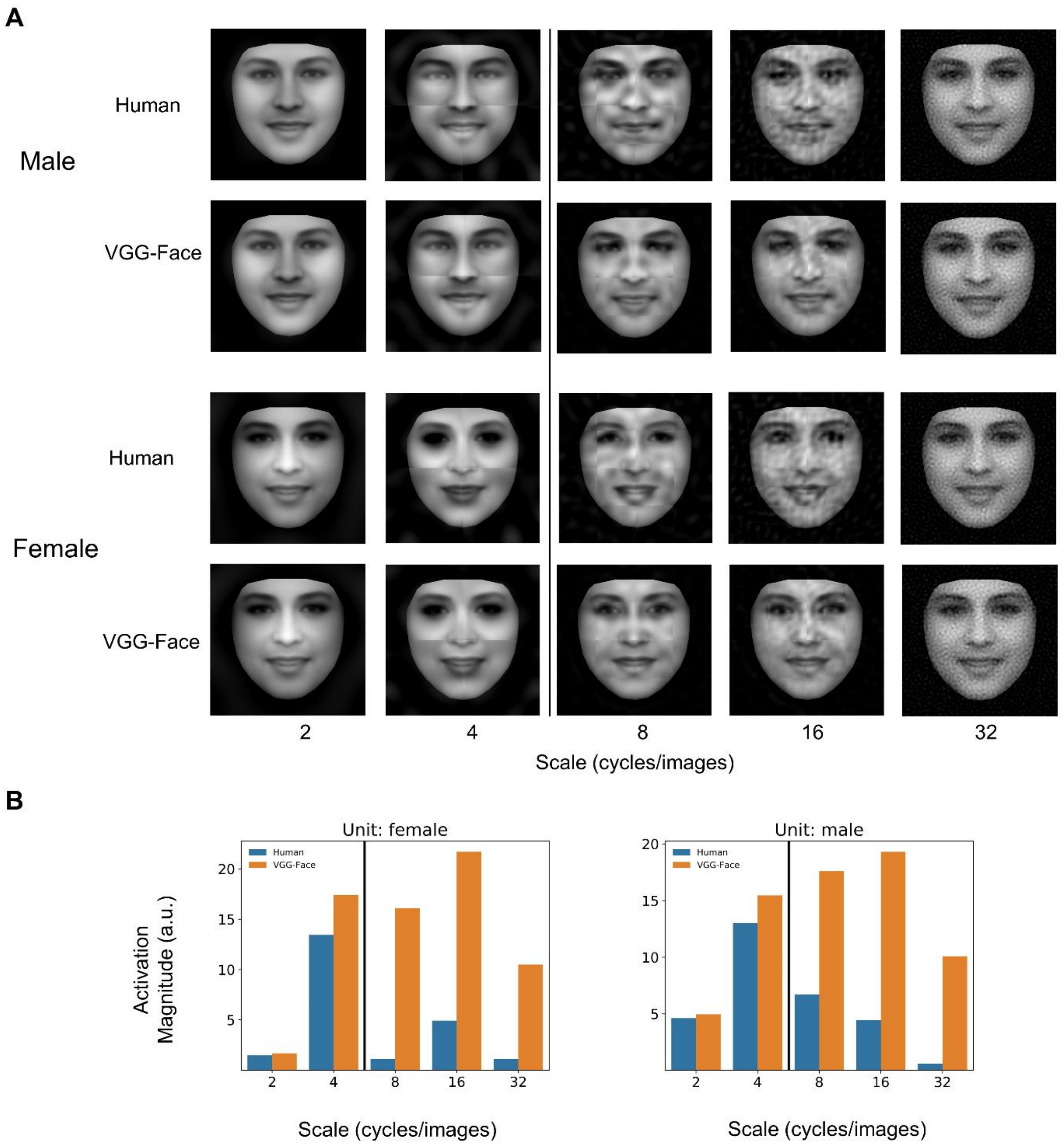

Supplementary Figure 1. Male and female prototypes at different scales. (a) male and female prototypes of the VGG-Face and human observers at different scales of spatial frequencies. (b) the activation magnitude of the units at the output layer of the VGG-Face (Left: female; Right: male).

Supplementary analysis 2

To examine the parameters that contributed differently between the VGG-Face and human observers, we first standardized the Cohen's d, the index for the size of contribution, to z-score for the VGG-Face and human observers respectively so thatwe can compare the contribution of the parameters across different scales. That is, the larger difference between the two z-scores of a parameter was, the more distinct contribution of the parameter between the VGG-Face and human observers. When the difference of a parameter deviated from the average contribution difference more than 1.96 standard deviations of the distribution of contribution differences, the difference was defined as significant. As shown in Figure S2a, the number of parameters that contributed significantly different between the VGG-Face and human observers differed. Further analyses revealed that the number of the parameters increased monotonically as a function of the scales of spatial frequencies, which were 3, 21, 40, 55, 64 at the scales of 2, 4, 8, 16, and 32 cycles/image respectively (Figure S2b). This result supplemented the finding that the VGG-Face and human observers made similar use of low spatial frequencies by showing that the difference in representation mainly came from face information at high spatial frequencies.

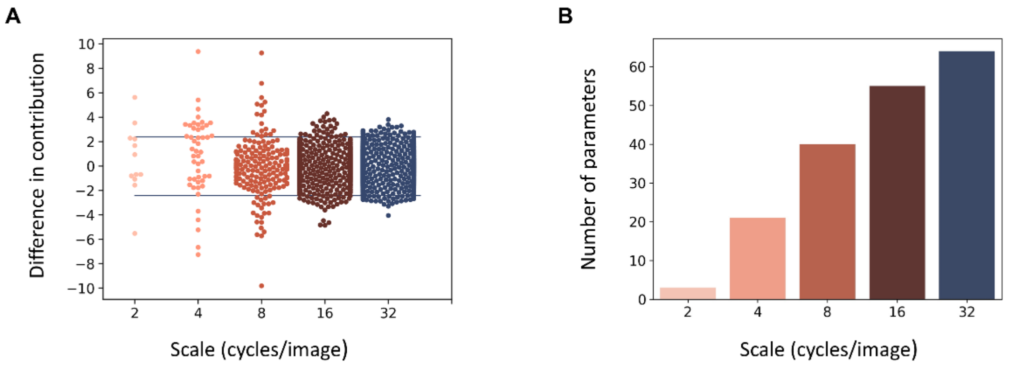

Supplementary Figure 2. (A) Distribution of the contribution difference (the difference between standardized Cohen's d in VGG-Face and humans) at different scales of spatial frequencies. The blue lines denote 1.96 standard deviations from the mean difference; **(B)** the number of parameters contributing significantly different between the VGG-Face and human observers at different scales of spatial frequencies.

Supplementary analysis 3

VGG-16 has the same architecture as the VGG-Face, but different prior task experience as it is pre-trained for object categorization as AlexNet. We trained the VGG-16 to perform the gender classification task with the same transfer learning procedure as that for the VGG-Face, and the testing accuracy of gender classification was 94%, indicating that it was able to perform the task. However, the CI obtained from the VGG-16 (Figure S3) was in sharp contrast to the CIs of human observers either as a whole ($r = 0.14$) or at different scales ($r = 0.44, -0.05, -0.09, 0.01$ and 0.0) at the scales of 2, 4, 8, 16 and 32). We also reconstructed female and male prototypes of VGG-16, and they appeared quite distinct from those of human observers and the VGG-Face (Figure 1c).

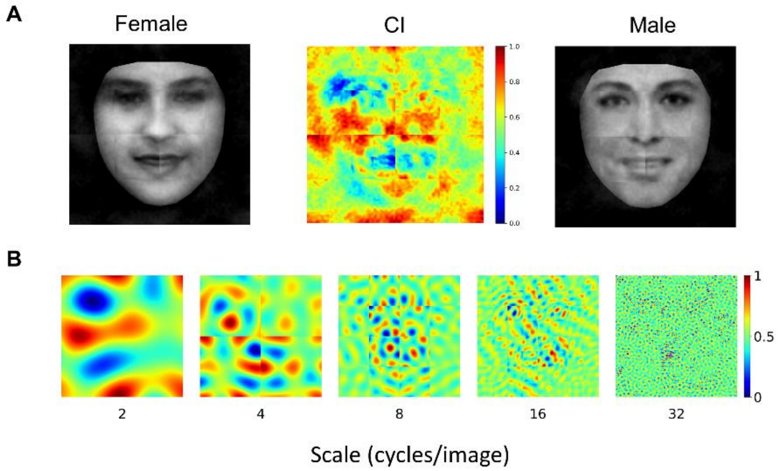

Supplementary Figure 3**.** Classification images of the VGG-16 as a whole **(A)** and at different scales of spatial frequencies **(B)**.

Supplementary analysis 4

Given that the training sample contained more male than female faces, we also trained the VGG-Face and AlexNet for face-gender classification with balanced training sample to exclude the possibility that our results was caused by unbalanced training sample. Specifically, 29000 face images (14500 females) of 65 identities from VGGFace2 were used as the training set, and 1000 faces (500 females) of the same 65 identities were used as the validation set. In addition, 1000 faces (500 females) of different 25 identities from VGGFace2 were used as the testing set. After training, for the VGG-Face, the accuracy for gender classification reached 97.9% on the training sample, 98.6% on the validation sample, and 98.5% on the testing sample. For the AlexNet, the accuracy reached 86.8% on the training sample, 92.4% on the validation sample, and 89.1% on the testing sample. After transfer learning, we presented template faces embedded in noises to VGG-Face and AlexNet and obtained CIs from them. The main findings were replicated, that is, the CI obtained from VGG-Face showed a much higher correlation with that obtained from humans ($r = 0.69$) than the correlation between the CIs from AlexNet and humans ($r = -0.13$). These results indicated that our findings were not caused by unbalanced training set or using faces of the same identities in the training and testing sets.

Supplementary analysis 5

To examine whether our results could transfer to other face databases, we trained the VGG-Face and AlexNet for face-gender classification using face images from another database, the Face Attribute Dataset for Balanced Race, Gender, and Age (ref). Specifically, 9954 faces (4666 females) from the Face Attribute Dataset were used as the training sample, and 500 faces (246 females) were used as the validation sample. Additionally, 1000 faces (500 females) from VGGFace2 were used as the testing sample. After training, for the VGG-Face, the accuracy for gender classification reached 85.3% on the training sample, 85.5% on the validation sample, and 99.1% on the testing sample. For the AlexNet, the accuracy reached 62.9% on the training sample, 61.8% on the validation sample, and

73.1% on the testing sample. After transfer learning, we presented template faces embedded in noises to VGG-Face and AlexNet and obtained CIs from them. Consistent with the main findings in manuscript, the CI obtained from VGG-Face showed a much higher correlation with that obtained from humans ($r = 0.62$) than the correlation between the CIs from AlexNet and humans ($r = 0.24$).

Supplementary Table 1. The confusion matrices of VGG-Face and AlexNet in testing after transfer learning of gender classification.

Network	True labels	Predicted labels	
		Female	Male
VGG-Face	Female	490	10
	Male	4	496
AlexNet	Female	422	78
	Male	29	471

Supplementary Table 2. The precision, recall, and F1-score of VGG-Face and AlexNet in testing after transfer learning of gender classification.

Network	Classes	Precision (%)	Recall (%)	F1-score (%)
VGG-Face	Female	99.2	98	98.6
	Male	98	99.2	98.6
AlexNet	Female	93.6	84.4	88.7
	Male	85.8	94.2	89.8
